# PopCover-2.0. Improved Selection of Peptide Sets With Optimal HLA and Pathogen Diversity Coverage

**DOI:** 10.3389/fimmu.2021.728936

**Published:** 2021-08-17

**Authors:** Jonas Birkelund Nilsson, Alba Grifoni, Alison Tarke, Alessandro Sette, Morten Nielsen

**Affiliations:** ^1^Department of Health Technology, Section for Bioinformatics, Technical University of Denmark, Lyngby, Denmark; ^2^Center for Infectious Disease and Vaccine Research, La Jolla Institute for Immunology, La Jolla, CA, United States; ^3^Department of Internal Medicine, University of Genoa, Genoa, Italy; ^4^Department of Experimental Medicine, University of Genoa, Genoa, Italy

**Keywords:** vaccine design, epitope selection, HLA, HLA class I, HLA class II, rational epitope selection, pathogen coverage, allelic coverage

## Abstract

The use of minimal peptide sets offers an appealing alternative for design of vaccines and T cell diagnostics compared to conventional whole protein approaches. T cell immunogenicity towards peptides is contingent on binding to human leukocyte antigen (HLA) molecules of the given individual. HLA is highly polymorphic, and each variant typically presents a different repertoire of peptides. This polymorphism combined with pathogen diversity challenges the rational selection of peptide sets with broad immunogenic potential and population coverage. Here we propose PopCover-2.0, a simple yet highly effective method, for resolving this challenge. The method takes as input a set of (predicted) CD8 and/or CD4 T cell epitopes with associated HLA restriction and pathogen strain annotation together with information on HLA allele frequencies, and identifies peptide sets with optimal pathogen and HLA (class I and II) coverage. PopCover-2.0 was benchmarked on historic data in the context of HIV and SARS-CoV-2. Further, the immunogenicity of the selected SARS-CoV-2 peptides was confirmed by experimentally validating the peptide pools for T cell responses in a panel of SARS-CoV-2 infected individuals. In summary, PopCover-2.0 is an effective method for rational selection of peptide subsets with broad HLA and pathogen coverage. The tool is available at https://services.healthtech.dtu.dk/service.php?PopCover-2.0.

## Introduction

T cell based vaccines and diagnostics offer an appealing alternative to their antibody based counterpart. T cell epitopes are short peptide fragments presented on the surface of cells in a complex with human leukocyte antigen (HLA) molecules. Since the peptides are short, careful selection can ensure high conservation between different pathogen strain variants, potentially resolving the issue of pathogen “escape” and antigenic drift observed towards antibody-driven vaccines and diagnostics. A key example of this includes the current issue of vaccine efficacy against SARS-CoV-2 variants. However, HLA molecules are extremely diverse with more than 29,000 allelic variants of the HLA genes currently annotated in the IMGT-HLA database (1). This immense diversity imposes a major challenge when designing T cell based vaccines and diagnostics, since each HLA molecule has a unique peptide binding specificity resulting in a potential to bind and present a unique set of antigenic peptides to the T cells of the host [reviewed in (2)]. Given this, T cell epitope characterization and T cell vaccine development is in many aspects the hallmark of personalized immunology. However, even though each HLA binds a unique peptide repertoire, peptides can be identified that bind promiscuously to multiple HLA. Identification of HLA specific peptidomes can be achieved with high accuracy for the vast majority of prevalent HLA class I and class II molecules using current state-of-the-art prediction methods for HLA peptide antigen presentation [reviewed in (3)], and such in-silico methods serve as integral components of most current rational epitope discovery pipelines [reviewed in (2)]. However, even limiting the analysis to a subset of prevalent HLA alleles and a likewise small representative set of pathogen genotypes, the exhaustive list of predicted HLA binders will in most cases end up entailing 1,000-100,000 peptides, and the task of selecting an optimal subset of these to include in a vaccine and/or diagnostic kit remains far from trivial. Many different approaches have been proposed to deal with the challenge such as Mosaic (4), OptiTope (5, 6), PopCover (7) and others (8, 9). While the Mosaic approach seeks to generate an artificial protein compiled from multiple pathogen derived peptides, yielding a high coverage of naturally occurring 9-mers without any consideration of potential for HLA antigen presentation, the other methods seek to identify peptide subsets in different manners providing optimal HLA and pathogen genotype coverage. In a benchmark study, Schubert et al. compared the performance of these different methods and demonstrated that methods that simultaneously aim to optimize pathogen and HLA coverage (i.e., OptiTope and PopCover) significantly outperformed methods focusing on pathogen coverage alone (i.e., Mosaic) (10). These different approaches have been applied with high success in both vaccine design (11) and for identification of broadly immunogenic peptide data sets (7). However, none of the current peptide driven tools allow an automatic and in depth approach to satisfy the important aspects of vaccine design associated with peptide redundancy and integration of CD4 and CD8 immunity. Here, we resolve these limitations by proposing an updated version 2.0 of the PopCover method allowing identification of peptide subsets from large data set(s) of predicted HLA class I and/or HLA class II binders with optimal HLA and pathogen genotype coverage, and showcase the power of this method on large protein data sets from HIV and SARS-CoV-2.

## Materials and Methods

### Allele Frequencies

Allele frequencies were obtained from the Allele Frequency Net Database (12).

### PopCover-2.0 Input Data - Peptide Binders and Protein Data

In PopCover-2.0, HLA class I and class II binder data are loaded separately, and the program can thus take either one or two input files with peptide-HLA binders. In a given input binder file, each line contains a peptide and an HLA allele, along with an optional genotype. A fourth optional column with the peptide’s binding core can also be included, which gives more specificity in regard to allele and genotype combinations.

An example of an HLA class I dataset is given below:

peptide_1_ allele_1_ genotype_1_ [core_1,1_]
peptide_1_ allele_2_ genotype_2_ [core_1,2_]
peptide_2_ allele_1_ genotype_2_ [core_2,1_]

Analyzing these lines results in the following annotation:

**Table:** 

**Peptide**	**Alleles**	**Genotypes**	**Allele, genotype combinations**
peptide_1_	allele_1_ allele_2_	genotype_1_ genotype_2_	(allele_1_, genotype_1_) (allele_2_, genotype_2_)
peptide_2_	allele_1_	genotype_2_	(allele_1_, genotype_2_)

In other words, data lines with the same peptide are combined into one entry in the final data structure, keeping track of all covered alleles, genotypes and their combinations. Binding cores are handled in the same way, thereby keeping track of allele, genotype, binding core combinations for each peptide. If no binding core column is provided, the peptides themselves are treated as their own binding core in the data structure.

A separate file containing the protein sequences from which the peptide binders originate can also be included. This file can either be in FASTA format or simply formatted with one protein sequence per line. If a protein sequence file is submitted, all overlapping peptides of a given length *n* will be extracted from the sequences, and the input HLA binder peptides are mapped onto them. During this mapping, any binder peptide which is a substring of a given *n*-mer peptide will pass on its HLA, genotype and binding core profile to the *n*-mer peptide. Any *n*-mer peptide not containing HLA binders is discarded afterwards. This will thus result in a list of unique *n*-mer peptides all covering certain allele, genotype combinations. On the other hand, if no protein sequence file is submitted, the initial peptide list will simply be the set of input binder peptides, which may all vary in length.

### Dataset Reduction

In large peptide datasets there is often some redundancy in the form of nested/sub-string peptides with overlapping HLA and genotype coverage. To deal with this redundancy, PopCover-2.0 uses a dataset reduction method that results in a list of unique peptides with low redundancy in terms of sequence and HLA, genotype profiles.

**Figure 1:**
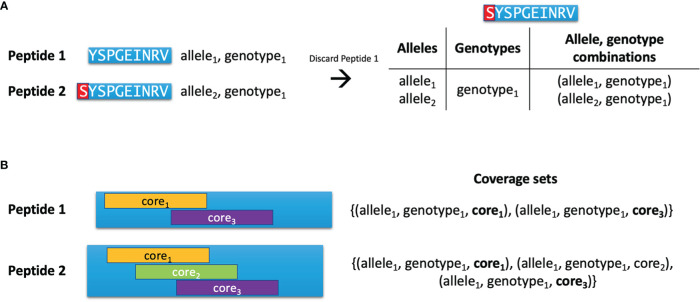
Pairwise comparison of peptides. **(A)** Peptide 1 is an HLA-binder which is contained as a substring in Peptide 2, which is also an HLA binder. Peptide 1 is thus discarded, and Peptide 2 inherits the alleles and genotypes covered by Peptide 1. **(B)** Two peptides are compared during the Hobohm 1 algorithm. Peptide 2 has three binding cores and covers one allele and one genotype, yielding three combinations in its coverage set. Peptide 1 has two of the same binding cores as Peptide 2, and covers the same allele and genotype as Peptide 2, giving it two of the same combinations as Peptide 2. As such, the coverage set of Peptide 1 is a subset of Peptide 2’s coverage set, and Peptide 1 is discarded due to it being functionally redundant.

After preparing the initial list of peptides with nested HLA binders, the list is reduced using the Hobohm 1 algorithm (13). This algorithm keeps a list of ‘unique’ peptides, and peptides are only added to this list if they are not deemed similar to any of the peptides already assigned to the unique list. Here, the similarity criterion depends on which types of peptides are being compared. If no input protein sequences were provided, it is possible for the two peptides being compared to have different lengths. Here, one peptide can be deemed redundant if it is a substring of the other peptide (see **Figure 1A**). In this case, the longer peptide ‘inherits’ the allele, genotype and binding core information from the shorter peptide. However, if this criterion is not fulfilled or the peptides have equal length, another criterion is used based on the peptides’ so-called **coverage sets**, which are sets of unique allele, genotype, binding core combinations (see **Figure 1B**). Each time a peptide is deemed functionally redundant due to it having a subset of the same coverage set combinations as another peptide, it is discarded.

The Hobohm 1 algorithm requires that peptides are sorted in descending order of importance to ensure that important peptides are more likely to be added to the unique peptide list. In PopCover-2.0, the peptide list is sorted first by length, then by the size of the coverage sets (i.e. the number of allele, genotype, binding core combinations in the sets). This results in the longest peptides and peptides with the widest allele, genotype profiles being at the top of the list.

### Allelic Coverage

To accurately calculate the allelic coverage of a set of peptide HLA binders, the concept of phenotypic frequency is utilized, given by the following equation:

$$q=2f-f^{2}$$

where *f* is the allelic frequency of a given allele. The phenotypic frequency is equal to the probability of observing a given allele within a population. This is due to the fact that an individual carries two HLA alleles per locus, and that these two alleles can be identical. If a given selection of epitopes covers *k* alleles within some locus, then the locus coverage is equal to

$$Coverage_{locus}=1-\overset{k}{\underset{i=1}{\Pi }}\left(1-q_{i}\right)$$

where *q_i_* is the phenotypic frequency of the *i*’th allele. After completing the coverage calculation for each locus individually within either HLA class I or HLA class II, the coverage across loci can be found by the following:

Coverage_across_ = Coverage_A_
Coverage_across_ += (1 - Coverage_across_) · Coverage_B_
Coverage_across_ += (1 - Coverage_across_) · Coverage_C_

where Coverage_across_ in this example is the coverage across the three HLA class I loci. The same type of calculation can then be carried out for HLA class II.

### Epitope Selection

To make an optimal selection of the first epitope, an initial coverage score is given to all eligible peptides, which is given by the following relation:

$$S_{ini}=\left(C_{1}+C_{11}\right)\;\cdot G$$

where C_I_ and C_II_ are the individual peptide’s coverage across HLA class I and HLA class II loci, respectively, and *G* is the number of pathogen genotypes covered by the peptide. The peptide with the highest initial score is thus selected as the first epitope. The remaining epitopes are then selected using the relation below

$$S_{J}^{A+G}=\sum_{i}\sum_{k}\frac{R_{ki}^{j}\cdot f_{k}\cdot g_{i}}{\beta +E_{ik}}$$

where $R_{ki}^{j}$ is 1 if epitope *j* derives from genotype *i* and binds to allele *k* and otherwise 0, *f_k_* is the allelic frequency of allele *k*, g*_i_* is the genomic frequency which is usually set as uniform, *E_ik_* is the number of times the combination of allele *k* and genotype *i* has been covered by the previously selected peptides, and *β* is a tunable constant which controls the emphasis on the individual peptide coverages as opposed to the uncovered allele, genotype combinations. By selecting the highest scoring peptide in each round, an optimal set of epitopes in terms of allele and genotype coverage is achieved.

### Experimental Validation of T Cell Response to Peptide Pools

The peptides selected in this study as well as additional sets of SARS-CoV-2 peptides previously identified experimentally (14) or by prediction with NetMHCpan 4.0 (15) were synthetized as crude material (TC Peptide Lab, San Diego, CA). All peptides were individually resuspended in dimethyl sulfoxide (DMSO), pooled according to composition and underwent subsequent lyophilization with final resuspension in DMSO at 1 mg/mL as previously reported (16). Flow-cytometry based activation induced cell marker (AIM) assay was performed on 10 COVID-19 convalescent donors (see **Supplementary Table A**), as previously reported (14).

Briefly, PBMCs were stimulated for 20-24 hours by either one of the different peptide pools (1 μg/ml) or with an equimolar amount of DMSO as negative control or with PHA (Roche, 1μg/ml) as positive control. After stimulation, cells were stained with CD3 AF700 (4:100; Life Technologies), CD4 BV605 (4:100; BD Biosciences), CD8 BUV496 (2:100; BD Biosciences), and Live/Dead eFluor506 (5:1000; eBioscience). To measure T cell activation the following markers were additionally included in the staining: CD137 APC (4:100; Biolegend), OX40 PE-Cy7 (2:100; Biolegend), and CD69 PE (10:100; BD Biosciences). All samples were acquired on a ZE5 5-laser cell analyzer (Bio-rad laboratories) and analyzed with FlowJo software (Tree Star).

The CD4+ and CD8+ T cell reactivity was measured by co-expression of CD137/OX40 or CD137/CD69 markers, respectively. The % of AIM+ cells in the negative controls were removed from each peptide pool and also used to calculate the Stimulation Index (% of AIM+ cells pools/negative control). We considered a peptide pool response to be positive when the stimulation index was greater than 2 and the background subtracted data was greater than 0.02% or 0.03% for CD4+ or CD8+ T cells, respectively.

## Results

### The Webserver

A freely available web-server implementation of the PopCover-2.0 method is implemented at: https://services.healthtech.dtu.dk/service.php?PopCover-2.0.

#### Submission Page

##### Data Submission

Three main fields for input data are included: MHC class I binders, MHC class II binders and allele frequencies. Instead of pasting data into these fields, input data can also be uploaded in individual text files. While allele frequencies are a required input, the user can upload either HLA class I or class II binders, or both types. As mentioned earlier, an option is available to also upload the set of protein sequences ‘hosting’ the set of HLA binding peptides. Similarly, these can either be pasted into the input field or uploaded in a text file. Submitting these will run PopCover-2.0 in an alternative ‘mode’ where peptides of a user-defined length are extracted from the sequences, onto which HLA binders are mapped. This approach is more thorough as the number of contained binders per peptide can be potentially much larger than when only the input HLA binders are considered, and more sequence information can thus go into the epitope selection.

A sample data set of HLA binders, allele frequencies and protein sequences can be inputted by clicking on the “Insert sample data” button. Furthermore, a button for submitting the input is included on the bottom of the page.

##### Extra Configuration

Various numerical options are available for customization. The default number of 5 epitopes to select can be changed, along with how many times this selection should be repeated (referred to as ‘number of epitope sets’). The desired peptide length to be extracted from the optional input protein sequences can also be changed. Furthermore, the parameter *β* of the PopCover scoring function can be adjusted, along with the minimum genomic coverage (i.e. the minimum fraction of genotypes that a peptide must cover for it to be considered).

The applied method for selecting peptides can also be changed from the default PopCover scoring method. An alternative method ranks the peptides based on their initial scores (S_ini_) and selects the top scoring peptides. Additionally, an option for random selection of peptides is included for benchmarking purposes.

Additionally, several binary options can be left checked or unchecked. If either of the HLA binder input data do not contain a binding core column, the corresponding option must be checked. The Hobohm 1 dataset reduction can be skipped entirely, which can result in more redundancy between the selected peptides, as well as fewer covered alleles if the HLA binders have not been mapped onto *n*-mer peptides extracted from submitted protein sequences. The more technical options include using phenotypic frequencies instead of allelic frequencies for *f_k_* in the peptide scoring, and subtracting the minimum genomic coverage from the PopCover score function denominator.

After adding input data and changing relevant options, the job can be submitted by clicking on the ‘Submit’ button.

#### Output Page

**Figure 2:**
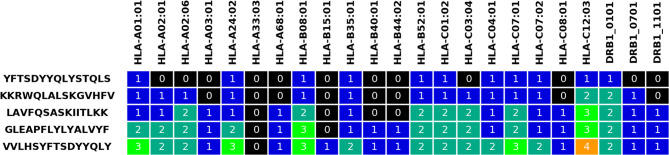
Visualization table for five selected peptides and the input HLA alleles. The grid numbers represent how many times a given allele has been covered by the current and previously selected peptides.

The output page first includes an overview of selected parameters, information on the submitted data before and after Hobohm 1 reduction, and an overview of all submitted alleles and genotypes. Afterwards, the selected peptides are presented with various statistics, along with the calculated allelic coverage values. Finally, an overview of the selected peptides can be downloaded in.txt format, and by default colored visualizations of the peptides are also available in.png and.xlsx formats (see **Figure 2**, similar visualization is provided for the genotype coverage). These visualizations illustrate the change in coverage of alleles and genotypes with each selected peptide.

### Evaluations

The functionalities and performance of the PopCover-2.0 method was showcased on two data sets; one from HIV and one from SARS-CoV-2.

#### HIV

Here, the dataset used in the original PopCover-1.0 publication (7) was re-applied using the updated PopCover-2.0 method. The dataset is composed of peptides from the 453 different HIV genotype Nef proteins, predicted using NetMHCII and NetMHCIIpan to bind one or more of 39 prevalent HLA-DRB1 molecules. The original publication also included information from HLA-DRB3, 4, 5 and HLA-DQ all assigned with low frequency. These predictions were excluded here to limit the calculations to cover only one locus. 15 epitopes were selected with PopCover-2.0 in order to compare with the same number of epitopes selected using PopCover-1.0. The two peptide selections are included in **Supplementary Table B**.

**Figure 3:**
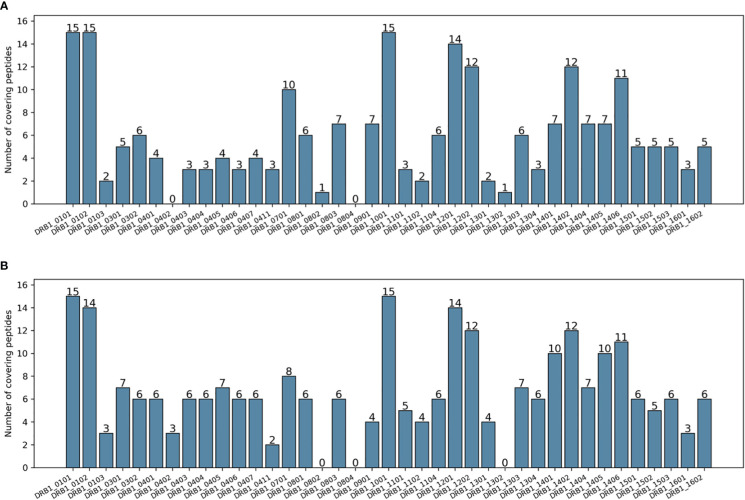
Number of peptides covering each DRB1 allele of the HIV dataset. **(A)** Peptides selected by PopCover-1.0. **(B)** Peptides selected by PopCover-2.0.

**Table 1** shows a series of calculated metrics for the epitope selections in terms of both allelic and genotypic coverage for the two methods. While PopCover-2.0 achieved a slightly lower DRB1 coverage and one allele fewer covered, it had a higher average number of peptides covering each allele (also illustrated in **Figure 3**). Additionally, in terms of genotypic coverage, PopCover-2.0 outperformed PopCover-1.0 by a very large margin, both in terms of number of covered genotypes and the average number of peptides per covered genotype.

**Table 1:** Coverage metrics of the peptides selected from the HIV dataset using PopCover-1.0 and PopCover-2.0.

	PopCover-1.0	PopCover-2.0
**DRB1 coverage**	0.869	0.856
**Number of alleles covered**	37 out of 39	36 out of 39
**Average number of peptides per covered allele**	6.2	7.2
**Number of genotypes covered**	160 out of 453	424 out of 453
**Average number of peptides per covered genotype**	1.06	2.44

#### SARS-CoV-2 Peptide-Based Vaccine

Having demonstrated that PopCover-2.0 achieves a comparable (and even slightly improved) performance compared to the original implementation for selection of peptides covering a single HLA loci and several pathogen genotypes, we next turned to demonstrate the truly new power of the updated tool; namely the ability to merge HLA class I and HLA class II predicted epitopes to identify peptide sets with optimal coverage of both antigen presentation pathways.

To showcase this, we used three proteins from the novel SARS-CoV-2 virus, namely the spike protein (S protein), nucleocapsid protein (N protein) and the ORF3a protein. These proteins are either candidates used in most current SARS-CoV-2 vaccines (S), or proteins suggested to be targets of immunodominant T cell responses (N, ORF3a) (17). Virus genomes were fetched from NCBI at (18) from which protein sequences were extracted. Sequences with missing or non-standard amino acids were excluded from the final dataset. An overview of the data used can be found in **Table 2**. From the set of unique protein sequences, all unique 8, 9 and 10-mer peptides were used as input to NetMHCpan 4.1 to predict binding to a set of 73 worldwide prevalent HLA class I alleles (identified from Allelefrequencies.net, and included in **Supplementary Table C**). Additionally, unique 15-mer peptides were extracted and used as input to NetMHCIIpan 4.0 for prediction of binders to 35 worldwide prevalent HLA-DRB1 alleles (also identified from Allelefrequencies.net, and included in **Supplementary Table C**.

**Table 2:** Overview of SARS-CoV-2 protein sequence data.

Protein ID	Number of unique protein sequences	Average number of unique 8, 9 and 10-mer peptides per unique protein sequence	Total number of unique 8, 9 and 10-mer peptides among unique protein sequences	Number of unique 9-mer peptides shared between all unique protein sequences
S protein	1724	3794.7	33051	18
N protein	1120	1232.9	16750	7
ORF3a protein	838	800.7	13634	0

From the HLA-I predictions, 32,374 strong (with %Rank less than or equal to 0.5%) HLA class I ligands were identified (10,526 for HLA-A, 12,338 for HLA-B, and 9,510 for HLA-C). For HLA class II, a total of 10,761 strong DRB1 ligands (with %Rank less than or equal to 1%) were predicted. These peptide binders were thus used as input for PopCover-2.0, together with allelic frequencies and the protein sequences for each of the three target proteins.

10 peptides were selected for each of the three proteins with PopCover-2.0 using three different selection methods, namely the standard (PopCover) method, random selection, and selection based only on initial scores S_ini_. Note here, that the random selection is limited to the set of HLA binding peptides remaining after redundancy reduction, and hence is performed on a set of peptides with overall high HLA binding potential (see **Supplementary Table D** for an overview of the peptides obtained before and after redundancy reduction). Further, an alternative selection based on the NetMHCpan (version 4.1) predictions was considered. Here, the peptides from the Wuhan-Hu-1 strain with the lowest %Rank towards each of the top 4 HLA-A, 4 HLA-B and 2 HLA-C alleles were selected (10 peptides selected in total for each protein, one for each HLA). Note that here, only HLA-I coverage was considered, as the peptides were not mapped onto longer peptides targeting the DRB1 allele. See **Supplementary Tables F**, **G** and **H** for the selected peptides using the three PopCover-2.0 methods and the NetMHCpan top 10 method.

**Figure 4:**
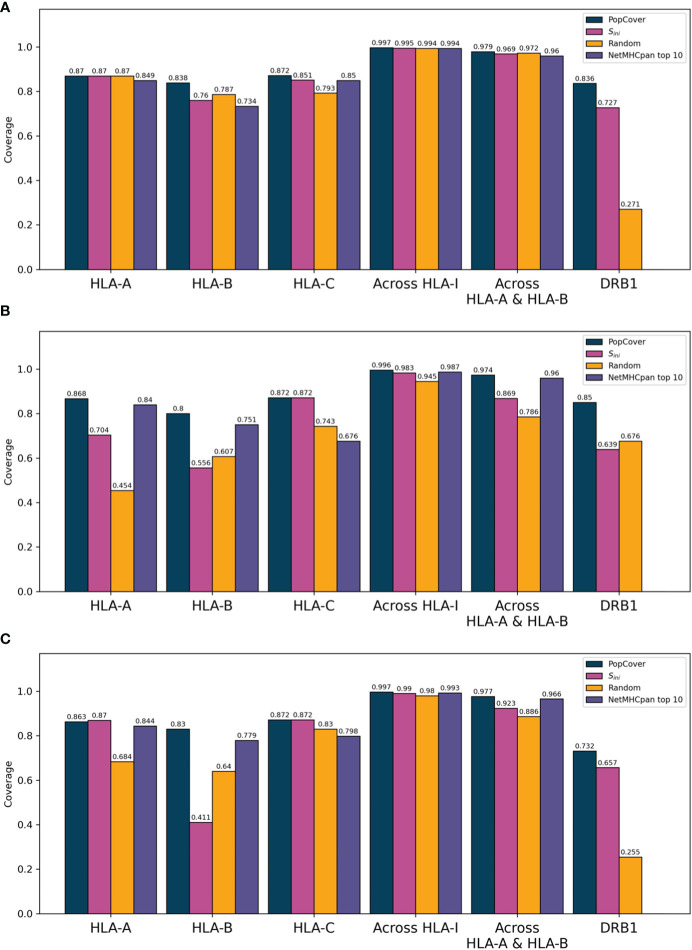
Population coverage of peptide sets selected by the PopCover, S_ini_, Random, and NetMHCpan top 10 methods for the three SARS-CoV-2 protein sets (**A**: S protein, **B**: N protein, **C**: ORF3a protein). Each bar chart shows the coverage estimated based on the three individual HLA-I loci (HLA-A, -B, and -C), the combined “across HLA-I” coverage, the coverage across only HLA-A and HLA-B, and lastly the HLA-II DRB1 locus. Note that the NetMHCpan top 10 peptides only target HLA-I alleles, hence no DRB1 coverage bars are included for this selection method.

For each selection method, the population coverage of the individual HLA loci was plotted (**Figure 4**). When investigating the population coverage of the selected peptides, the S_ini_ selection yielded an overall lower HLA class I and class II coverage compared to that obtained by PopCover. As expected, the random selection had very mixed population coverage across the three proteins, and displayed an overall much worse coverage in the different loci compared to the two other methods. On the other hand, the NetMHCpan top 10 selection yielded a high HLA class I coverage close to the level of the PopCover selection. As the HLA-C locus is more conserved and HLA-C alleles thus overall have higher frequencies than the other HLA-I loci (19), its coverage was observed to be close to even between the selection methods across all proteins (**Figure 4**). Because of this, an across-loci HLA-I coverage excluding HLA-C was included, which indicates a larger difference in HLA-I population coverage between the selection methods when controlling for the more conserved HLA-C locus (**Figure 4**).

**Figure 5:**
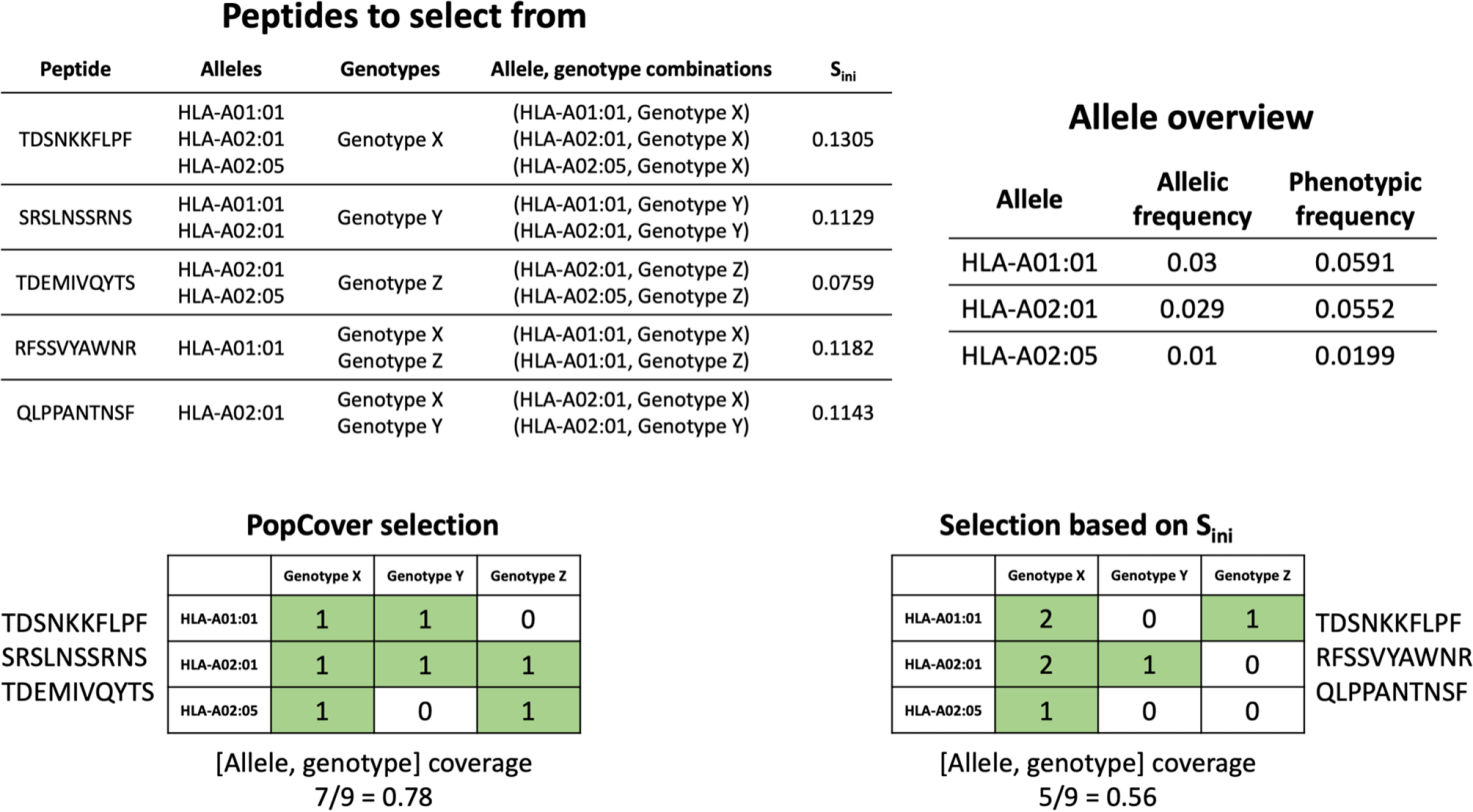
Example of allele, genotype coverage calculation for a selection of three peptides using both the PopCover selection method and selection based only on S_ini_. The grid numbers in the selection matrices indicate how many times each combination of allele and genotype has been covered by the selected peptides.

**Figure 6:**
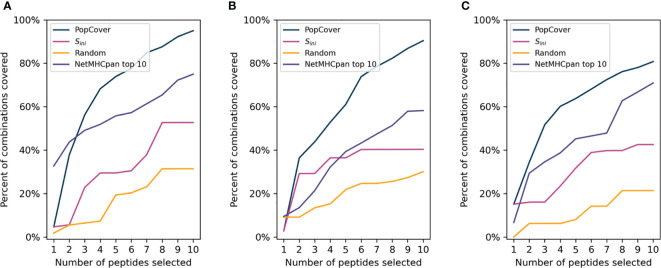
Accumulative allele, genotype combination coverage of peptides selected by the PopCover, S_ini_, Random, and NetMHCpan top 10 methods for the three SARS-CoV-2 protein datasets (**A**: S protein, **B**: N protein. **C**: ORF3a protein). For the PopCover, S_ini_ and Random methods the percentages are calculated on the combined HLA-I and HLA-II allele sets, while the percentages for theNetMHCpan top 10 method are calculated on the HLA-I allele set only.

In order to assess the different selection methods’ abilities to optimize both population and pathogen genotype coverage, the accumulative percent coverage of all possible <HLA, genotype> combinations for the different selection methods was calculated. **Figure 5** illustrates how the selection method can affect this metric, with the coverage of peptides identified using two selection methods, PopCover and S_ini_, being illustrated. In this example, the two selection methods both end up having full coverage of all alleles and genotypes. However, the S_ini_ selection method based on the initial coverage score results in coverage of fewer <HLA, genotype> combinations compared to the PopCover selection method. This example thus illustrates how individual peptide coverage does not necessarily lead to optimal coverage in the 2D space of HLA, genotype combinations. This observation is further showcased in **Figure 6**, when analyzing coverage of the peptide sets identified using the PopCover, S_ini_, random and NetMHCpan top 10 selection schemes for the three proteins. The results in **Figure 6** demonstrate the strong power of the PopCover selection method for selecting peptides that fill the 2D coverage space. In contrast, as shown for instance for the coverage curves for the N protein (**Figure 6B**), little to no improvement in HLA, genotype combination coverage is observed with the S_ini_ method after six selected peptides, as the S_ini_ method ends up choosing peptides with similar individual coverage. As expected, the random selection does not optimize the number of combinations covered, and, the NetMHCpan top 10 method achieves a reasonably high 2D coverage as the Wuhan strain from which the peptides were selected is the ancestor to the other included genotypes.

### Immunogenic Potential of Selected Peptides

In order to verify the immunogenic potential of the selected peptides, they were compared to a list of known SARS-CoV-2 epitopes from the IEDB (as of date: 22/4/2021). Only epitopes without post-translational modifications were considered, and lengths were restricted to between 8 and 13 for Class I and between 13 and 19 for Class II to include only likely minimally mapped epitopes. Further, only epitopes with two or more positive assays were included resulting in a set of 424 CD8 and 296 CD4 epitopes. A minimum substring match of length eight and nine was used as a criterion for whether or not a peptide selected by PopCover-2.0 had overlap with an experimentally verified CD8 and CD4 epitope, respectively. For the IEDB, the set of assay-verified SARS-CoV-2 epitopes with either MHC class I restriction or MHC class II restriction were used.

**Figure 7:**
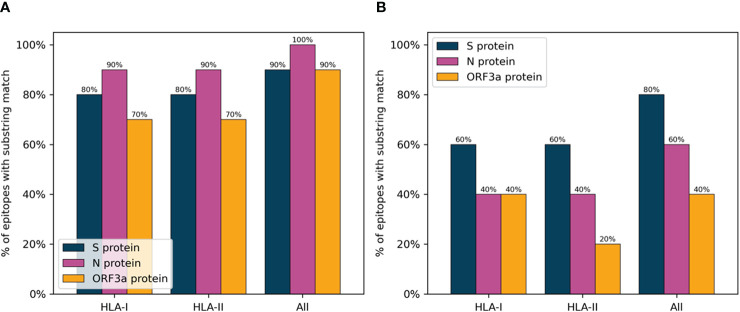
Percentage of peptides selected by the PopCover **(A)** and Random **(B)** methods with substring match to IEDB SARS-CoV-2 epitopes. HLA-I: Percent overlap calculated on HLA-I epitopes, with a substring length of 8 or more amino acids. HLA-II: Percent overlap calculated on HLA-II epitopes, with a substring length of 9 or more amino acids. All: Percent overlap calculated on the combined set of HLA-I and HLA-II epitopes, with minimum substring lengths of 8 and 9 amino acids for HLA-I and HLA-II epitopes, respectively.

**Figure 8:**
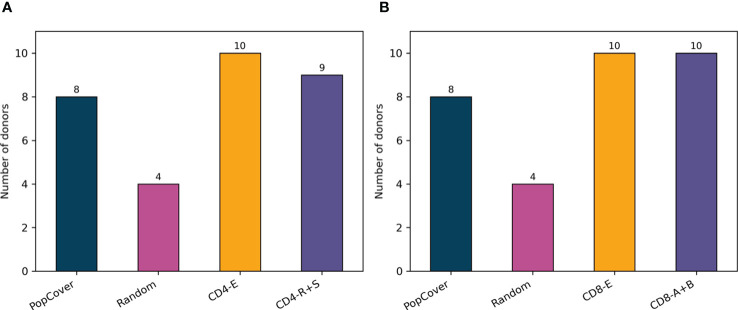
Number of donors with CD4 **(A)** or CD8 **(B)** response to each peptide pool tested, based on measurements with background subtracted. PopCover: 15-mers selected by the PopCover method (10 peptides from each of the proteins S, N and ORF3a, N = 30). Random: 15-mers randomly selected (10 peptides from each of the proteins S, N and ORF3a, N = 30). CD4-E and CD8-E: experimentally derived epitopes from Tarke et al. (14). CD4-R+S: 15-mers overlapping by 10 spanning the spike protein combined with the 7-allele predicted epitopes based on the remainder of the SARS-CoV-2 proteome (N = 221 for R and N = 253 for S). CD8-A+B: peptide pool in the top 1 percentile selected by NetMHCpan 4.0 after prediction with 12 HLA class I alleles with Pf>6% (N = 618).

**Figure 7** displays the results of this analysis, confirming a large overlap between verified SARS-CoV-2 epitopes and the peptides selected with the PopCover selection method. In comparison, the randomly selected peptides, as expected, consistently displayed a lower overlap with positive epitopes. However, since the list of tested SARS-CoV-2 peptides remains limited and highly biased towards predicted HLA binders (14), the fact that the randomly selected peptides have a lower percentage overlap to known epitopes does not necessarily mean that the random peptides are not immunogenic; they simply might not yet have been tested experimentally. To investigate this, the peptide selections using the PopCover and Random methods were tested experimentally for T cell response in a panel of SARS-CoV-2 infected individuals (for details refer to materials and methods). These experiments confirmed the increased immunogenic potential of the PopCover selected peptides (**Figure 8**). For both the CD4 and CD8 response assays, the Random peptides elicited an immune response in only 40% (4 out of 10) donors, while the PopCover peptides gave a response in 80% (8 out of 10) donors. In comparison, the CD4 and CD8 peptide megapools described by Tarke et al. (14) gave responses in 80-100% of the tested donors. Note, these results should further be interpreted in the context of the PopCover selected peptide sets only covering the S, N and ORF3a SARS-CoV-2 proteins, in contrast to the megapools that were selected from the entire SARS-CoV-2 proteome.

One donor had neither CD4 or CD8 response to the PopCover peptide pool, while two donors had only a CD4 or CD8 response, respectively. The HLA profiles of these three donors were investigated in regard to their HLA allelic frequencies, and how these compared to the HLA frequencies of the seven other donors with both CD4 and CD8 response to the PopCover peptide pool. In each of the two groups of donors, the list of alleles of each donor which were considered in the PopCover analysis were concatenated into a shared allele list. Here, it was found that the alleles of the uncovered donors had significantly lower mean allelic frequency compared to those of the remaining donors, *p*=0.022, two-sample t-test (see **Supplementary Table E**). This suggests that the three donors without full response to the PopCover peptide pool had uncommon HLA profiles, resulting in a lower HLA restriction overlap to the selected peptide set, and further highlights the critical importance of an accurate HLA frequency characterization in order to select population covering peptide sets.

## Discussion

T cell epitopes constitute a promising alternative to traditional whole antigen approaches for vaccine design, therapeutics and diagnostics development. This has been illustrated in several contexts including cancer T cell immunotherapy (20, 21) and SARS-CoV-2 diagnostics (examples include (14, 22–24)]. As T cell receptors are specific to peptide-HLA complexes, T cell responses are most often highly patient (imposed by HLA diversity) and pathogen (imposed by genotype diversity) individualized, challenging the development of broadly applicable T cell based therapeutics and diagnostics. Here, we have demonstrated how the simple PopCover approach can be used to address this challenge. Using HIV and SARS-CoV-2 as examples, PopCover-2.0 was demonstrated to allow for effective selection of small peptide pools with broad HLA and genotype coverage, allowing for the identification of patient and pathogen diversity agnostic peptide pools.

Other approaches for optimal peptide pools have been suggested including epitope megapools to facilitate identification and quantification of SARS-CoV-2-specific CD4+ and CD8+ T cells responses (14, 25, 26). While such megapools containing thousands of individual peptides have proven highly successful for the bulk quantification and functional characterization of T cell responses in infected individuals, the approach is highly cost-intensive and might not be universally applicable. Further, the approach has limited applicability in the context of T cell epitope based vaccine design, where the possible number of included peptides is highly limited.

In contrast to earlier approaches for rational design of optimal T cell epitope-based vaccines (and diagnostics), the PopCover-2.0 method proposed here allows for identification of peptides with coverage of CD4 and CD8 immunogenicity. The power of this unique feature was demonstrated in the context of COVID-19 where the pools of peptides identified from three SARS-CoV-2 proteins were demonstrated to induce both CD4 and CD8 responses in the vast majority of tested COVID-19 positive individuals. In line with what has been observed earlier for instance Yellow fever (27) and cancer neo-epitopes (28, 29) the ability to target both arms of the cellular immune system is essential for the induction of potent and effective vaccine responses. Given this, we believe this unique feature of PopCover-2.0 makes it a powerful complement to other vaccine design strategies.

## Data Availability Statement

The datasets presented in this study can be found in online repositories. The names of the repository/repositories and accession number(s) can be found in the article/**Supplementary Material**.

## Author Contributions

JN developed the PopCover-2.0 software and wrote the *Results* section along with the parts of the *Materials and Methods* section concerning the PopCover-2.0 implementation. AG contributed towards the *Materials and Methods* sections regarding the experimental part of the study and organizing the experiments. AT carried out the experiments described in the paper. AS contributed towards organizing the experiments. MN wrote the sections *Abstract*, *Introduction*, and *Discussion*. All authors contributed to the article and approved the submitted version.

## Funding

This work was supported in part by US Federal funds from the National Institute of Allergy and Infectious Diseases, National Institutes of Health, Department of Health and Human Services, under Contract No. HHSN272201200010C to MN and contract No. 75N9301900065 and 75N93019C00001 to AS. AT was supported by a PhD student fellowship through the Clinical and Experimental Immunology Course at the University of Genoa, Italy.

## Conflict of Interest

The authors declare that the research was conducted in the absence of any commercial or financial relationships that could be construed as a potential conflict of interest.

## Publisher’s Note

All claims expressed in this article are solely those of the authors and do not necessarily represent those of their affiliated organizations, or those of the publisher, the editors and the reviewers. Any product that may be evaluated in this article, or claim that may be made by its manufacturer, is not guaranteed or endorsed by the publisher.
