# Supplementary material for: PopCover-2.0. Improved Selection of Peptide Sets With Optimal HLA and Pathogen Diversity Coverage
Source: Front Immunol. 2021 Aug 17;12:728936. doi: 10.3389/fimmu.2021.728936 (PMC8416060; doi:10.3389/fimmu.2021.728936)
Supplement: Supplementary file 1 [file DataSheet_1.docx]

Availability of data and materials

The datasets supporting the conclusions of this article are available in the GitHub repository, <https://github.com/JonasNilsson1/popcover-2.0>.

# Supplementary tables

**Supplementary Table A**. Characteristics of donor cohorts.

|  | **COVID-19 (n = 10)** |
| --- | --- |
| **Age** (years) | 20-67 [Median = 38, IQR = 31] |
| **Gender**  Male (%)  Female (%) | 60% (6/10)  40% (4/10) |
| **Sample Collection Date** | May–Sept 2020 |
| **SARS-CoV-2 PCR** | Positive = 100% (6/6)  Not tested= 40% (4/10) |
| **S RBD IgG Positive** | 90% (9/10) |
| **Peak disease Severity^a^**  Mild  Moderate | 60% (6/10)  40% (4/10) |
| **Race-Ethnicity**  White- not Hispanic  Asian | 90% (9/10)  10% (1/10) |
| **Days at Collection**^b^ | 66-140 [Median = 101, IQR = 87] |

^a^According to WHO criteria.
^b^Post Symptom Onset

**Supplementary Table B:** Peptide selections on the HIV dataset by PopCover-1.0 and PopCover-2.0.

| **PopCover-1.0** | **PopCover-2.0** |
| --- | --- |
| AVDLSHFLKEKGGLM  EVLMWKFDSRLAFHH  EVLMWKFDSRLALTH  EVLVWRFDSRLAFHH  FPVRPQVPLRPMTYR  GKWSKSSIVGWSAVR  GLDGLIYSKKRQEIL  GWCFKLVPVDPDEVE  GWCFKLVPVEPEEVE  GWFLKEKGGLDGLIY  GWPFKLVPVDPREVQ  KWSKNRIVGWPAVRE  QVPLRPMTYKGALDL  RWEFDSSLARRHLAR  SWFLKEKGGLEGLIY | DLSHFLKEKGGLEGL  DWQNYTPGPGVRYPL  EVLKWQFDSSLARRH  EVLMWKFDSRLAFHH  EVLVWKFDSRLAFHH  FGWCFKLVPVDPREV  GFPVRPQVPLRPMTY  GLIYSKKRQEILDLW  KHGAITSSNTAATNA  LIYSRKRQEILDLWV  LSFFLKEKGGLDGLI  LTFGWCFKLVPVDPD  WKFDSSLARRHLARE  WKFDSSLARRHMARE  WSKSSIVGWPAVRER |

**Supplementary Table C:** Alleles and their frequencies used in the PopCover-2.0 SARS-CoV-2 analysis, fetched from Alellefrequencies.net

| **HLA-A** | **HLA-B** | **HLA-C** | **DRB1** |
| --- | --- | --- | --- |
| HLA-A01:01 0.1193  HLA-A02:01 0.247  HLA-A02:05 0.0076  HLA-A02:06 0.0106  HLA-A02:07 0.0086  HLA-A03:01 0.1167  HLA-A11:01 0.0733  HLA-A23:01 0.0259  HLA-A24:02 0.1024  HLA-A25:01 0.0321  HLA-A26:01 0.0379  HLA-A29:02 0.0191  HLA-A30:01 0.0201  HLA-A30:02 0.009  HLA-A31:01 0.0263  HLA-A32:01 0.0266  HLA-A33:01 0.0077  HLA-A33:03 0.0179  HLA-A66:01 0.006  HLA-A68:01 0.0314  HLA-A68:02 0.0099 | HLA-B07:02 0.0966  HLA-B08:01 0.0761  HLA-B13:02 0.0353  HLA-B14:02 0.0159  HLA-B15:01 0.0714  HLA-B15:02 0.0096  HLA-B15:03 0.0061  HLA-B15:18 0.006  HLA-B18:01 0.049  HLA-B27:02 0.01  HLA-B27:05 0.0322  HLA-B35:01 0.0522  HLA-B35:02 0.0083  HLA-B35:03 0.0223  HLA-B37:01 0.0107  HLA-B38:01 0.0221  HLA-B39:01 0.0135  HLA-B40:01 0.0474  HLA-B40:02 0.0309  HLA-B41:02 0.0082  HLA-B44:02 0.0533  HLA-B44:03 0.0407  HLA-B45:01 0.0071  HLA-B46:01 0.0101  HLA-B49:01 0.0126  HLA-B50:01 0.0092  HLA-B51:01 0.0453  HLA-B52:01 0.0156  HLA-B53:01 0.0111  HLA-B55:01 0.0101  HLA-B56:01 0.0098  HLA-B57:01 0.0264  HLA-B58:01 0.015 | HLA-C01:02 0.0512  HLA-C02:02 0.0593  HLA-C03:02 0.0089  HLA-C03:03 0.0498  HLA-C03:04 0.068  HLA-C04:01 0.118  HLA-C05:01 0.0521  HLA-C06:02 0.1024  HLA-C07:01 0.1322  HLA-C07:02 0.131  HLA-C07:04 0.021  HLA-C08:01 0.0086  HLA-C08:02 0.021  HLA-C12:02 0.0155  HLA-C12:03 0.0761  HLA-C14:02 0.0134  HLA-C15:02 0.0204  HLA-C16:01 0.0191  HLA-C17:01 0.0135 | DRB1_0701 0.095725  DRB1_1501 0.079008  DRB1_0301 0.072938  DRB1_1101 0.059725  DRB1_0101 0.048581  DRB1_0901 0.045288  DRB1_1302 0.043978  DRB1_1301 0.042651  DRB1_1502 0.038644  DRB1_0405 0.034716  DRB1_1202 0.030382  DRB1_0401 0.030040  DRB1_1104 0.026471  DRB1_1401 0.025283  DRB1_0803 0.023349  DRB1_1201 0.022978  DRB1_0403 0.020916  DRB1_0404 0.020473  DRB1_1001 0.017347  DRB1_0802 0.016259  DRB1_1602 0.015587  DRB1_0102 0.015284  DRB1_1503 0.014835  DRB1_1601 0.014789  DRB1_0407 0.014421  DRB1_1303 0.011286  DRB1_0801 0.010942  DRB1_1402 0.010527  DRB1_0804 0.009089  DRB1_0402 0.008593  DRB1_0302 0.008567  DRB1_0406 0.008291  DRB1_1102 0.007167  DRB1_1404 0.006084  DRB1_1405 0.006058 |

**Supplementary Table D**: Overview of the peptide pools obtained before and after applying Hobohm 1 redundancy reduction.

|  | **Number of unique 15-mer peptides** | | **Average number of covered alleles per peptide** | | **Average number of HLA, genotype, [binding core] combinations per peptide** | |
| --- | --- | --- | --- | --- | --- | --- |
| **Before/after redundancy reduction** | **Before** | **After** | **Before** | **After** | **Before** | **After** |
| **S protein** | 7398 | 2980 | 3.1 | 6.0 | 555.9 | 1301.5 |
| **N protein** | 3021 | 1403 | 3.2 | 5.2 | 253.2 | 522.1 |
| **ORF3a protein** | 3140 | 1416 | 3.3 | 5.8 | 149.3 | 328.5 |

**Supplementary Table E:** Summary statistics of allele frequencies of donors grouped based on response to the PopCover peptide pool.

|  | **Mean allele frequency** | **Median allele frequency** | **Allele frequencies standard deviation** |
| --- | --- | --- | --- |
| Alleles among donors without CD4 and/or CD8 response to PopCover peptides | 0.059 | 0.043 | 0.042 |
| Alleles among donors with both CD4 and CD8 response to PopCover peptides | 0.09 | 0.075 | 0.06 |

**Supplementary Table F1:** Peptide selection using the PopCover method from the SARS-CoV-2 S protein dataset.

| **Peptide** | **Accumulative number of covered alleles** | **Accumulative number of covered genotypes** | **Accumulative % coverage of (allele, genotype) combinations** |
| --- | --- | --- | --- |
| EKGIYQTSNFRVLPT | 37 | 1718 | 0.04645 |
| YYVGYLQPRTFLLKY | 53 | 1724 | 0.37767 |
| GEVFNATRFASVYAW | 71 | 1724 | 0.56248 |
| TSNFRVQPTESIVRF | 80 | 1724 | 0.68267 |
| LTDEMIAQYTSALLA | 83 | 1724 | 0.73886 |
| AIPTNFTISVTTEIL | 86 | 1724 | 0.7757 |
| NTQEVFAQVKQIYKT | 92 | 1724 | 0.84856 |
| EPQIITTDNTFVSGN | 95 | 1724 | 0.87619 |
| GTHWFVTQRNFYEPQ | 100 | 1724 | 0.92212 |
| NDILSRLDKVEAEVQ | 103 | 1724 | 0.94981 |

**Supplementary Table F2:** Peptide selection using the S_ini_ method from the SARS-CoV-2 S protein dataset.

| **Peptide** | **Accumulative number of covered alleles** | **Accumulative number of covered genotypes** | **Accumulative % coverage of (allele, genotype) combinations** |
| --- | --- | --- | --- |
| EKGIYQTSNFRVLPT | 37 | 1718 | 0.04645 |
| VEKGIYQTSNFRVLP | 38 | 1718 | 0.05561 |
| PRRVRSVASQSIIAY | 53 | 1724 | 0.22858 |
| TQEVFAQVKQIYITP | 64 | 1724 | 0.29484 |
| HRRVRSVASQSIIAY | 64 | 1724 | 0.29487 |
| PPAYINSFTRGVYYP | 67 | 1724 | 0.30494 |
| TRVVYYPDKVFRSSV | 80 | 1724 | 0.3791 |
| TSNFRVQPTESIVRF | 85 | 1724 | 0.52693 |
| TSNFRIQPTESIVRF | 85 | 1724 | 0.52717 |
| NSFTRVVYYPDKVFR | 85 | 1724 | 0.52717 |

**Supplementary Table F3:** Peptide selection using the Random method from the SARS-CoV-2 S protein dataset.

| **Peptide** | **Accumulative number of covered alleles** | **Accumulative number of covered genotypes** | **Accumulative % coverage of (allele, genotype) combinations** |
| --- | --- | --- | --- |
| NKSWMESEFRAYSSA | 14 | 1663 | 0.01791 |
| EVFSQVKQIYKTPPI | 31 | 1724 | 0.05485 |
| LFLPFFSNVTLFHAI | 44 | 1724 | 0.06415 |
| DPFLGVYYHKYNKSW | 45 | 1724 | 0.0731 |
| NCYFPLQSYGFQPTY | 52 | 1724 | 0.1933 |
| NKLDSKVGGNYNYLY | 52 | 1724 | 0.20297 |
| SFIVEKGIYQTSNFR | 52 | 1724 | 0.23085 |
| NVTRFASVYAWNRKR | 60 | 1724 | 0.31404 |
| PHGVVFLHVNYVPAQ | 69 | 1724 | 0.3141 |
| TNGTKRFNNPVLPFN | 70 | 1724 | 0.31411 |

**Supplementary Table F4:** Peptide selection using the NetMHCpan top 10 method from the SARS-CoV-2 S protein dataset.

| **Peptide** | **Accumulative number of covered alleles** | **Accumulative number of covered genotypes** | **Accumulative % coverage of (allele, genotype) combinations** |
| --- | --- | --- | --- |
| YLQPRTFLL | 24 | 1714 | 0.32686 |
| LTDEMIAQY | 32 | 1724 | 0.43659 |
| RLFRKSNLK | 36 | 1724 | 0.49113 |
| QYIKWPWYI | 38 | 1724 | 0.51869 |
| SPRRARSVA | 41 | 1724 | 0.55766 |
| TLDSKTQSL | 42 | 1724 | 0.57223 |
| VLKGVKLHY | 45 | 1724 | 0.6134 |
| QELGKYEQY | 48 | 1724 | 0.65399 |
| VRFPNITNL | 53 | 1724 | 0.72245 |
| VYDPLQPEL | 55 | 1724 | 0.74971 |

**Supplementary Table G1:** Peptide selection using the PopCover method from the SARS-CoV-2 N protein dataset.

| **Peptide** | **Accumulative number of covered alleles** | **Accumulative number of covered genotypes** | **Accumulative % coverage of (allele, genotype) combinations** |
| --- | --- | --- | --- |
| HIDAYKTFPPTELKK | 35 | 1105 | 0.0282 |
| TKAYNVTQAFGRRGP | 51 | 1120 | 0.36423 |
| SASAFFGMSRIGMEV | 56 | 1120 | 0.43829 |
| VGMEVTPSGTWLTYT | 66 | 1120 | 0.52855 |
| KHIDAYKTFPPTEPK | 68 | 1120 | 0.61016 |
| KDQVILLNKHIDAYK | 82 | 1120 | 0.73881 |
| SPRWYFYYLGTGPEA | 87 | 1120 | 0.78502 |
| YTGAIKLDDKDPNFK | 91 | 1120 | 0.82283 |
| PKGFYAEGSRGGSQA | 95 | 1120 | 0.8683 |
| NKDGIIWVATEGALN | 99 | 1120 | 0.90418 |

**Supplementary Table G2:** Peptide selection using the S_ini_ method from the SARS-CoV-2 N protein dataset.

| **Peptide** | **Accumulative number of covered alleles** | **Accumulative number of covered genotypes** | **Accumulative % coverage of (allele, genotype) combinations** |
| --- | --- | --- | --- |
| HIDAYKTFPPTELKK | 35 | 1105 | 0.0282 |
| RTAIKAYNVTQAFGR | 54 | 1120 | 0.29196 |
| GTWFTYTGAIKLDDK | 62 | 1120 | 0.29236 |
| TKAYNVTQAFGRRGP | 65 | 1120 | 0.36472 |
| AKAYNVTQAFGRRGP | 65 | 1120 | 0.36477 |
| NKHIDAYKTFPSTEP | 67 | 1120 | 0.40256 |
| TKVYNVTQAFGRRGP | 69 | 1120 | 0.40279 |
| IKAYNVTQAFGRRGP | 69 | 1120 | 0.40283 |
| TKSYNVTQAFGRRGP | 69 | 1120 | 0.40302 |
| NKHIDAYKTFPLTEP | 70 | 1120 | 0.40328 |

**Supplementary Table G3:** Peptide selection using the Random method from the SARS-CoV-2 N protein dataset.

| **Peptide** | **Accumulative number of covered alleles** | **Accumulative number of covered genotypes** | **Accumulative % coverage of (allele, genotype) combinations** |
| --- | --- | --- | --- |
| GTLLTYTGAIKLDDK | 16 | 1104 | 0.09132 |
| TYTGAIKLGDKDPNF | 17 | 1104 | 0.09133 |
| QASSRSSSRSRNSPR | 22 | 1120 | 0.13459 |
| SKGFYAEGSRGGSQA | 30 | 1120 | 0.15278 |
| HQVILLNKHIDAYKT | 45 | 1120 | 0.21782 |
| PDDQIGYYRRATRRI | 48 | 1120 | 0.24639 |
| MAGNGGDAAFALLLL | 55 | 1120 | 0.2465 |
| TPKDHIGTRNPVNNA | 58 | 1120 | 0.25549 |
| DHIGTRNPANNVAIV | 60 | 1120 | 0.27335 |
| QALPHRQKKQQTVTL | 66 | 1120 | 0.30083 |

**Supplementary Table G4:** Peptide selection using the NetMHCpan top 10 method from the SARS-CoV-2 N protein dataset.

| **Peptide** | **Accumulative number of covered alleles** | **Accumulative number of covered genotypes** | **Accumulative % coverage of (allele, genotype) combinations** |
| --- | --- | --- | --- |
| LLLDRLNQL | 7 | 1105 | 0.09461 |
| SSPDDQIGYY | 10 | 1120 | 0.13519 |
| KTFPPTEPK | 16 | 1120 | 0.21356 |
| QFAPSASAF | 24 | 1120 | 0.32208 |
| FPRGQGVPI | 30 | 1120 | 0.39311 |
| QKKQQTVTL | 33 | 1120 | 0.43261 |
| AQFAPSASAF | 36 | 1120 | 0.47394 |
| MEVTPSGTW | 39 | 1120 | 0.51349 |
| QRNAPRITF | 44 | 1120 | 0.57871 |
| KHIDAYKTF | 44 | 1120 | 0.5819 |

**Supplementary Table H1:** Peptide selection using the PopCover method from the SARS-CoV-2 ORF3a protein dataset.

| **Peptide** | **Accumulative number of covered alleles** | **Accumulative number of covered genotypes** | **Accumulative % coverage of (allele, genotype) combinations** |
| --- | --- | --- | --- |
| LAVFYSASKIITLKK | 43 | 812 | 0.15111 |
| YFTSDYYQLYSTQLS | 52 | 838 | 0.34412 |
| KIVDEPEEHVQIHTI | 65 | 838 | 0.51729 |
| ATIPIQASLPFGWLI | 69 | 838 | 0.60143 |
| KKRWQLALSKGVHFV | 71 | 838 | 0.63775 |
| SASKIITLKKRWQLA | 76 | 838 | 0.68098 |
| TFFIYNKIVDEPEEH | 81 | 838 | 0.72495 |
| TSDYYQLYSTQLSTD | 85 | 838 | 0.76127 |
| VVLHSYFTSDYYKLY | 88 | 838 | 0.7801 |
| HVTFFIYNKIVDEPE | 91 | 838 | 0.80779 |

**Supplementary Table H2:** Peptide selection using the S_ini_ method from the SARS-CoV-2 ORF3a protein dataset.

| **Peptide** | **Accumulative number of covered alleles** | **Accumulative number of covered genotypes** | **Accumulative % coverage of (allele, genotype) combinations** |
| --- | --- | --- | --- |
| LAVFYSASKIITLKK | 43 | 812 | 0.15111 |
| TSDYYQLYSTRLSTD | 52 | 838 | 0.1606 |
| TSDYYQLYPTQLSTD | 54 | 838 | 0.16081 |
| TSDYYQLYSTQLSID | 55 | 838 | 0.23414 |
| FTSDYYQLYSAQLST | 60 | 838 | 0.31749 |
| TSDYYQLYSTQLSTD | 60 | 838 | 0.3887 |
| QSASKIITLKKRRQL | 67 | 838 | 0.39753 |
| TSDYYQLYSTQLNTD | 67 | 838 | 0.39776 |
| HSASKIITLKKRLQL | 67 | 838 | 0.42548 |
| ASDYYQLYSTQLSTD | 67 | 838 | 0.42558 |

**Supplementary Table H3:** Peptide selection using the Random method from the SARS-CoV-2 ORF3a protein dataset.

| **Peptide** | **Accumulative number of covered alleles** | **Accumulative number of covered genotypes** | **Accumulative % coverage of (allele, genotype) combinations** |
| --- | --- | --- | --- |
| VFQSASKILTLKKRW | 28 | 1 | 0.00031 |
| TSGDDTTSPISEHDY | 36 | 810 | 0.06207 |
| NPVMEPMYDEPTTTT | 37 | 810 | 0.0621 |
| ATPPDFVRATATIPI | 41 | 810 | 0.06214 |
| KIITFKKRWQLALSK | 43 | 838 | 0.08029 |
| TQLSTDTGVEHVTLF | 46 | 838 | 0.14198 |
| TSFDGTTSPISEHDY | 47 | 838 | 0.142 |
| SEHDYQIGGYTEKWE | 52 | 838 | 0.21342 |
| KIITLKKRRQLALSK | 53 | 838 | 0.21349 |
| SHLLLVAAFLEAPFL | 55 | 838 | 0.2136 |

**Supplementary Table H4:** Peptide selection using the NetMHCpan top 10 method from the SARS-CoV-2 ORF3a protein dataset.

| Peptide | Accumulative number of covered alleles | Accumulative number of covered genotypes | Accumulative % coverage of (allele, genotype) combinations |
| --- | --- | --- | --- |
| LLYDANYFL | 5 | 812 | 0.06637 |
| FTSDYYQLY | 22 | 837 | 0.29396 |
| SASKIITLK | 26 | 837 | 0.34626 |
| VYFLQSINF | 29 | 838 | 0.38699 |
| IPIQASLPF | 34 | 838 | 0.45186 |
| TLKKRWQL | 35 | 838 | 0.46515 |
| VLHSYFTSDY | 36 | 838 | 0.4785 |
| EEHVQIHTI | 47 | 838 | 0.62721 |
| CRSKNPLLY | 50 | 838 | 0.6678 |
| YYQLYSTQL | 53 | 838 | 0.70926 |
